# Effect of Vitamin D on the Proliferation and Barrier of Atrophic Vaginal Epithelial Cells

**DOI:** 10.3390/molecules28186605

**Published:** 2023-09-13

**Authors:** Dandan Li, Tao Zhang, He Yang, Wenlan Yang, Chi Zhang, Guolan Gao

**Affiliations:** 1 Savaid Medical School, University of Chinese Academy of Sciences, Beijing 101408, China; 2 School of Clinical Medicine, Gannan Medical University, Ganzhou 341004, China; 3 Department of Orthopedics, Peking University International Hospital, Beijing 102200, China; 4 Biomedical Engineering Department, Peking University, Beijing 100871, China

**Keywords:** vitamin D, ovariectomy, vaginal epithelium, tight junction proteins, atrophic vaginitis

## Abstract

Atrophic vaginitis is very common in postmenopausal women due to declining estrogen levels. Vitamin D plays an important role in promoting epithelial cell proliferation, migration and adhesion. We established a rat model of ovariectomy (OVX) induced atrophic vaginitis with the aim of investigating the effects of Vitamin D supplementation on the vaginal epithelial barrier. The results showed that ovariectomised rats had significantly higher vaginal pH, reduced *Lactobacillus*, significantly lower uterine and vaginal weights, and lower vaginal epithelial *PCNA*, *occludin*, and *E-cadherin* mRNA expression compared with sham-operated rats. Vitamin D supplementation could reduce the vaginal pH, promote the proliferation and keratinization of vaginal epithelial cells, enhance the expression of *PCNA* mRNA in vaginal tissues, and improve the vaginal and uterine atrophy. Vitamin D can also increase the expression of E-cadherin and occludin proteins in vaginal tissues, maintain the integrity of the vaginal epithelium, increase the number of *Lactobacillus*, and reduce pathogenic bacterial infections. In vitro experiments demonstrated that 1,25(OH)_2_D_3_ could promote the proliferation and migration of VK2/E6E7 vaginal epithelial cells and increase the expression of E-cadherin protein. In conclusion, we demonstrated that Vitamin D can regulate the expression of vaginal epithelial tight junction proteins, promotes cell proliferation, and improves vaginal atrophy due to estrogen deficiency.

## 1. Introduction

Atrophic vaginitis, also known as age-related vaginitis, is one of the most common problems in postmenopausal women [1]. By 2030, there will be approximately 1.2 billion postmenopausal women worldwide [2]. As estrogen production decreases in women after menopause, vaginal tissues experience atrophy, thinning of the mucosa, and decreased elasticity, leading to increased pain and itching during sexual intercourse [3]. At the same time, atrophy of the vaginal wall also leads to a decrease in defence ability, a decrease in the number of *Lactobacillus* bacteria, and an increase in the pH, causing pathogens to colonise the vagina, making it easy to develop vaginitis and urinary tract infections [4,5]. HPV infection is more likely to occur after menopause due to reduced local vaginal immunity [6]. Clinically, estrogen supplementation is mainly used to promote vaginal squamous epithelial proliferation. However, there is still a great deal of controversy regarding the long-term use of estrogen in postmenopausal women. Systemic hormone replacement therapy increases the risk of bleeding, breast cancer, endometrial cancer and cardiovascular disease. Topical hormone use does not increase any of these. However, not all postmenopausal women can receive hormone therapy [7]. Therefore, it is important to find safe and effective estrogen replacement therapy for atrophic vaginitis.

Vitamin D is a fat-soluble vitamin whose primary role is to promote the absorption of calcium and phosphorus from the mucosal cells of the small intestine, promote new bone production and calcification, and improve muscle strength [8]. Calcitriol is the main active form of vitamin D. Generally, vitamin D supplementation in humans is cholecalciferol. In recent years, there has been increasing interest in the role of Vitamin D beyond bone health. There is no consensus on serum 25(OH)D levels in patients with atrophic vaginitis. The Endocrine Society considers 25(OH)D concentrations below 30 ng/mL as Vitamin D insufficient. The Institute of Medicine considers 25(OH)D levels below 20 ng/mL to be vitamin D deficient [9,10]. Vitamin D receptor (VDR) are widely distributed in cells of various tissues in the body, and Vitamin D binding to VDR affects the expression of a number of genes. VDR are found in the basal cell layer of vaginal tissue [11] Vitamin D is involved in the growth and differentiation of vaginal epithelial cells and promotes maturation of the vaginal epithelium. Kamronrithisorn et al., found that weekly administration of 40,000 IU of Vitamin D to postmenopausal women resulted in a significant improvement in vaginal maturation indices and dryness symptoms [12]. A double-blind clinical trial demonstrated that topical Vitamin D vaginal suppositories lowered vaginal pH and improved vaginal dryness [13]. Abban et al., demonstrated in an ovariectomised rat model that Vitamin D induced an increase in vaginal epithelial cells, with effects consistent with corifin β [14]. In addition to topical estrogens, Vitamin D has a protective effect in women with atrophic vaginitis.

Tight junctions (TJs) proteins are the apical-most complexes of epithelial and endothelial cells, which are mainly composed of claudin, occludin, periplasmic family of proteins (ZO proteins), and junctional adhesion molecules [15]. TJs proteins play an important function in the maintenance of the integrity of the epithelial barrier. When tight junction proteins are reduced, barrier function is lost [16]. Vaginal microecology is a combination of endocrine regulation, normal flora in the vagina and an intact vaginal barrier [17]. The vagina is constantly exposed to a variety of pathogens and an intact vaginal structure reduces female reproductive tract infections. Vitamin D/VDR signalling regulates the expression of proteins such as claudin, ZO and occludin in TJs proteins, which are important for maintaining the mucosal barrier [18]. Vitamin D was found to further maintain intestinal epithelial barrier function [19], prevent renal fibrosis [20], prevent pulmonary fibrosis and airway remodelling [21], accelerate healing of epidermal injuries [22], and reduce urinary tract infections [23], through the regulation of tight junction proteins in multiple organs. Currently, there are more studies on Vitamin D regulation of intestinal epithelial TJs proteins, and little is known about the role of Vitamin D in vaginal tissue TJs proteins.

In this study, we established an ovariectomised rat model to simulate atrophic vaginitis. We demonstrated for the first time that Vitamin D can regulate the expression of vaginal epithelial tight junction proteins at the gene level and protein level, and promote the proliferation of vaginal epithelial cells, which is protective against vaginal atrophy in postmenopausal women. Moreover, Vitamin D is inexpensive and has no adverse effects, so Vitamin D supplementation could be a potential option for reducing menopausal symptoms caused by atrophic vaginitis.

## 2. Results

### 2.1. Effect of Vitamin D on Uterine and Vaginal Tissues of Ovariectomised Rats

**Figure 1:**
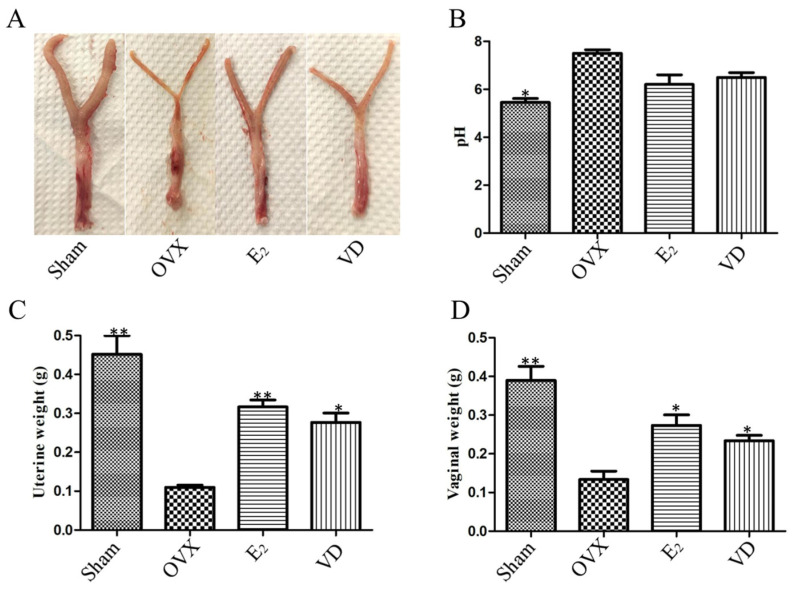
Vaginal and uterine morphological characteristics (**A**). Intravaginal pH (**B**). Uterine tissue weight (**C**). Vaginal tissue weight (**D**). Data are the mean ± SD (n = 6). * *p* < 0.05 and ** *p* < 0.01 compared to the OVX group.

The rats showed vaginal and uterine atrophy after ovariectomy, which was linear, well demarcated from the surrounding tissues, with poor haematopoiesis, and the cervix was soft and difficult to palpate (Figure 1A), which indicated the success of the modelling. Compared with the ovariectomy (OVX) group, the vaginal pH of rats in the sham surgery (Sham) group was reduced (*p* < 0.05), and there was a tendency for the pH to decrease in the estrogen (E_2_) and Vitamin D (VD) groups, but there was no statistical difference (Figure 1B). Compared with the Sham group, the uterine weight of rats in the OVX group was significantly reduced (*p* < 0.01) by about 75%. Compared with the OVX group, the E_2_ group reversed uterine atrophy and increased uterine weight, with a significant difference (*p* < 0.01), and Vitamin D supplementation similarly increased uterine weight by approximately 50% (Figure 1C). Vaginal weight was also significantly reduced after ovariectomy in rats. Compared with the Sham group, the vagina of OVX rats was significantly atrophied (*p* < 0.01) and weighed 68% less (Figure 1D). Supplementation with estrogen and Vitamin D increased vaginal weight (*p* < 0.05). It indicated that Vitamin D supplementation could reduce vaginal pH and improve vaginal uterine atrophy in ovariectomised rats.

### 2.2. Effect of Vitamin D on Hormone Levels in Ovariectomised Rats

**Figure 2:**
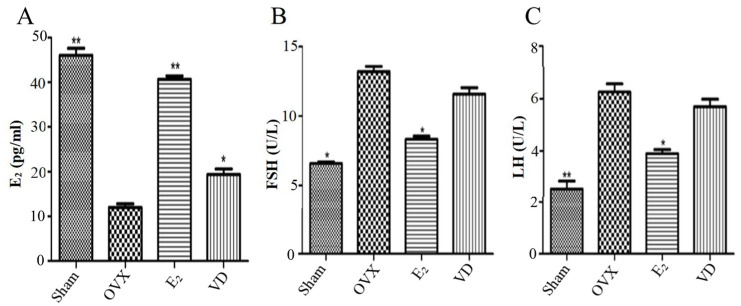
Serum sex hormone levels. (**A**) E_2_ levels, (**B**) FSH levels, (**C**) LH levels. Data are the mean ± SD of six data. Compare to the OVX group, * *p* < 0.05 and ** *p* < 0.01.

In order to investigate the effects of post Vitamin D on serum sex hormones in ovariectomised rats, we examined serum estrogen, follicle-stimulating hormone (FSH) and luteinising hormone (LH) levels. As shown in Figure 2, the serum estrogen levels of rats in the OVX group were significantly lower than those in the Sham and E_2_ groups (*p* < 0.01), and, FSH levels and LH levels were significantly higher than those in the Sham and E_2_ groups (*p* < 0.05). It indicated that the hormonal changes after successful modelling were similar to normal postmenopausal hormone levels. Compared with the OVX group, Vitamin D supplementation elevated estrogen levels (*p* < 0.05), but the difference in serum LH and FSH levels in the VD group was not statistically significant, indicating that Vitamin D supplementation did not affect FSH and LH levels in ovariectomised rats.

### 2.3. Effect of Vitamin D on Vaginal and Uterine Morphology in Rats

**Figure 3:**
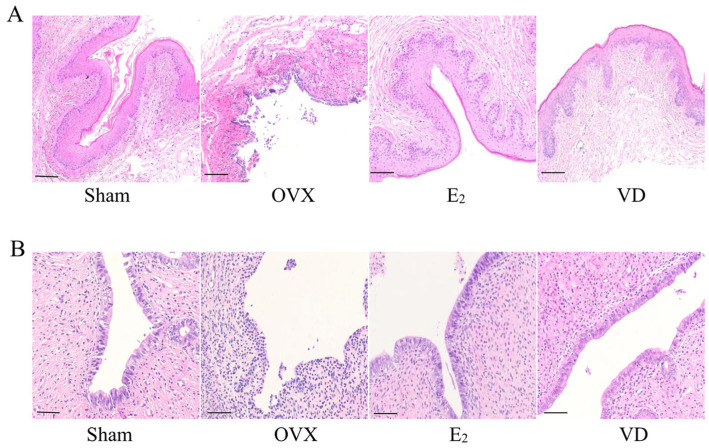
HE staining analysis of uterine and vaginal tissue. Scale bar = 100 µm. (**A**) HE analysis of vaginal tissue. (**B**) HE analysis of uterine tissue.

We observed the morphological changes of vagina and uterus by H&E staining. As shown in Figure 3A, the vaginal epithelium of the Sham group was structurally intact and thick, and the complex squamous epithelium with keratinisation was seen microscopically. The lamina propria was richly vascularised and was a dense connective tissue. The vaginal epithelium in the OVX group was atrophic with only 2–3 layers of cells thick and no keratinisation was seen. There were limited or diffuse defects in the mucosa with a large infiltration of inflammatory cells. Compared with the OVX group, the basal epithelial cells of the vagina of rats in the E_2_ and VD groups were proliferated, and significantly thickened complex squamous epithelium with intact structure was seen. This indicates that Vitamin D supplementation has the same effect as estrogen supplementation, both of which can increase the thickness of the vaginal wall and the integrity of the vaginal epithelium. We further observed the endometrial histomorphological changes in each group. As shown in Figure 3B, endometrial structure was normal in the Sham group, with tightly arranged single-layer columnar epithelium, thickening of the myometrium, and increased number of glands. The OVX group had uterine atrophy and thinning of the endometrium. The E_2_ group and the VD group were able to partially reverse uterine atrophy, promote secretion of mucus by endometrial glandular epithelial cells, and increase the thickness of the myometrium, which maintained the structural integrity of the endometrium.

### 2.4. Effect of Vitamin D on Gene Expression in Vaginal Tissues

**Figure 4:**
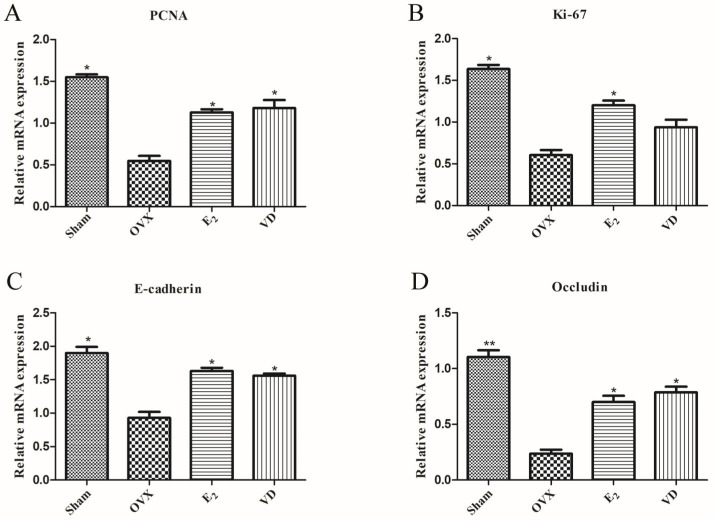
The mRNA expression of *PCNA* (**A**), *Ki-67* (**B**), *E-cadherin* (**C**) and *occludin* (**D**) in rat vaginal tissues was detected using qRT-PCR. Data are the mean ± SD (n = 6). Compared to the OVX group, * *p* < 0.05 and ** *p* < 0.01.

PCNA and Ki-67 are markers for evaluating the proliferative state of cells. Occludin and E-cadherin proteins are present in epithelial cells and maintain inter-epithelial cell adhesion and the structural and functional integrity of epithelial tissues. To investigate the effects of Vitamin D on the proliferation and intercellular adhesion of vaginal epithelial cells in ovariectomised rats, we examined the expression of proliferation and adhesion genes in rat vaginal tissues by qPCR. As shown in Figure 4A,B, *PCNA* and *Ki-67* mRNA expression was reduced in the OVX group compared with the Sham group (*p* < 0.05), indicating that the proliferative capacity of vaginal epithelial cells was decreased after ovariectomy in rats. Supplementation of estrogen and Vitamin D could increase the expression of *PCNA* at the gene level (*p* < 0.05), but the effect of Vitamin D supplementation on the expression of *Ki-67* mRNA was not obvious. After ovariectomy in rats with epithelial atrophy and mucosal defects, *E-cadherin* and *occludin* mRNA expression was decreased in the OVX group compared with the Sham group (Figure 4C,D). The E_2_ and VD groups could increase the expression of these two tightly linked proteins (*p* < 0.05). The results indicated that Vitamin D treatment could increase the expression of *PCNA*, *E-cadherin* and *occludin* mRNA, promote the proliferation of vaginal epithelial cells, and maintain the integrity of the vaginal barrier in ovariectomised rats.

### 2.5. Effect of Vitamin D on Microorganisms in the Rat Vagina

**Figure 5:**
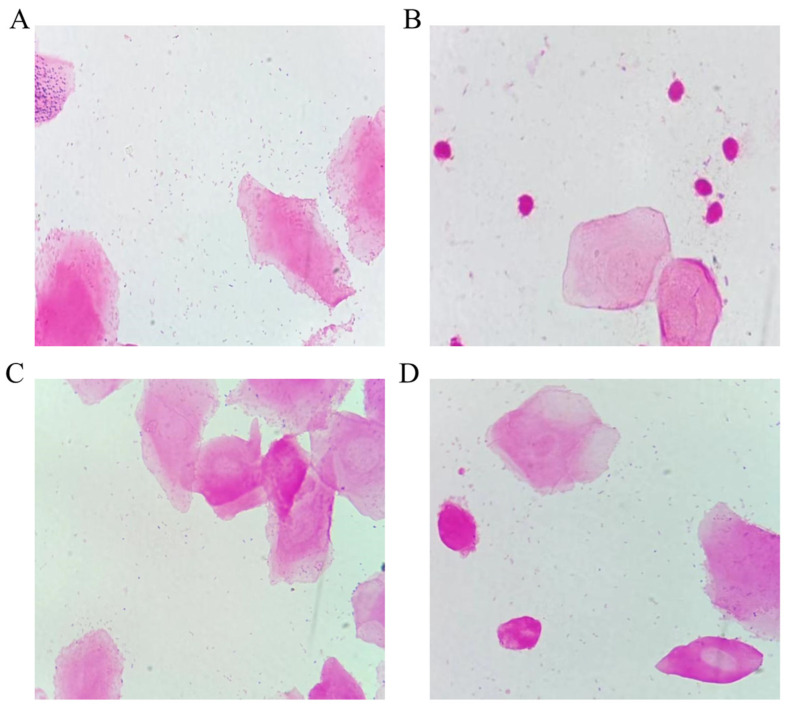
Gram stain analysis of vaginal bacterial smears (100×). (**A**) Sham group, (**B**) OVX group, (**C**) E_2_ group and (**D**) Vitamin D group.

Vaginal mucosal defects increase the number of harmful bacteria in the vagina and increase the infection rate of vaginitis in postmenopausal women. As shown in Figure 5A, after Gram staining of vaginal secretions in the Sham group, the vaginal epithelial cells were polygonal, and a large number of rod-shaped *Lactobacillus* bacteria were seen in the vaginal secretions, which showed normal vaginal microecology. A smear of secretions from rats in the OVX group showed a significant decrease in the number of *Lactobacillus* bacteria, a higher number of leukocytes, and stained portion of the impurities of vaginal secretions (Figure 5B). After supplementation of estrogen and Vitamin D, the vaginal secretion smears of rats in the E_2_ and VD groups showed a significant increase in the number of *Lactobacillus.* A large number of keratinised or unkeratinised epithelial cells, and fewer inflammatory cells are seen (Figure 5C,D). Due to the increase in the number of vaginal *Lactobacillus*, there was also a tendency for the vaginal pH of rats in the E_2_ and VD groups to decrease compared to the OVX group (Figure 1B).

### 2.6. Effect of Vitamin D on the VK2/E6E7 Cell Line

**Figure 6:**
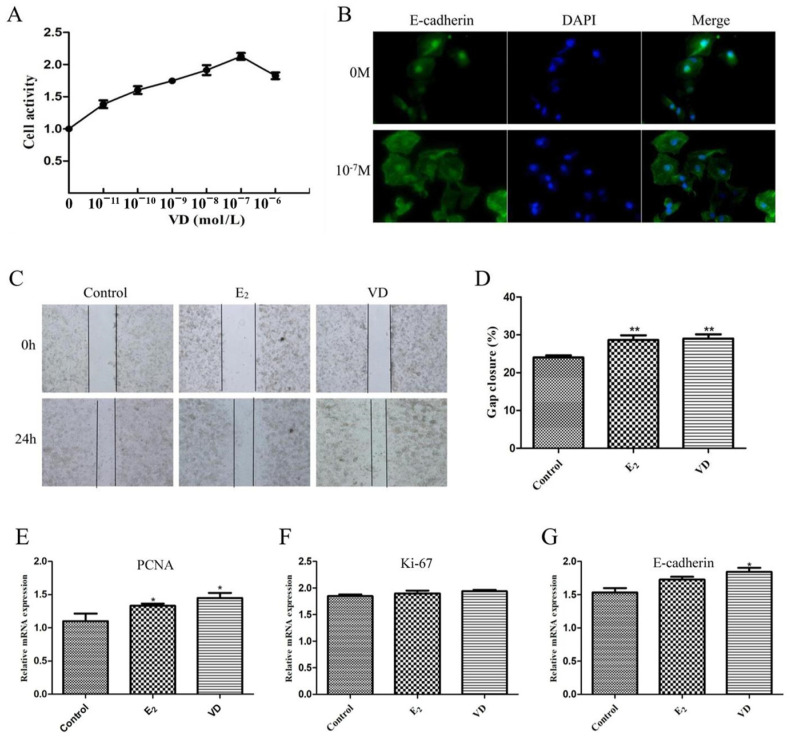
Effect of Vitamin D on proliferation, migration and adhesion of VK2/E6E7 cells. CCK 8 analysis of VK2/E6E7 cell proliferation (**A**). Immunofluorescence staining was analysed for E-cadherin protein (**B**). Scratch test to detect migration of VK2/E6E7 cells (**C**). Quantitative analysis of the migration ability of VK2/E6E7 cells (**D**). The mRNA expression levels of *PCNA* (**E**), *Ki-67* (**F**) and *E-cadherin* (**G**) in VK2/E6E7 cells were measured by qRT-PCR. Data are the mean ± SD (n = 6). * *p* < 0.05 and ** *p* < 0.01 compared to the OVX group.

We evaluated the effects of 1,25(OH)_2_D_3_ on the proliferation, migration and adhesion of VK2/E6E7 vaginal epithelial cells by in vitro experiments. Cell Counting Kit-8 (CCK8) experiments were performed to detect the effects of different concentrations of 1,25(OH)_2_D_3_ (10^−11^–10^−6^ M) on the viability of VK2/E6E7 cells. It was found that the cell viability was highest when 1,25(OH)_2_D_3_ concentration was 10^−7^ M and VK2/E6E7 cells were cultured for 24 h (Figure 6A). The expression of E-cadherin protein in VK2/E6E7 cells was next detected by immunofluorescence staining. VK2/E6E7 cells were cultured with 0 M and 10^−7^ M 1,25(OH)_2_D_3_ for 24 h, respectively, and it was found that the expression of E-cadherin protein in VK2/E6E7 cells was significantly increased by Vitamin D supplementation (Figure 6B). The migration of VK2/E6E7 cells by estrogen and 1,25(OH)_2_D_3_ was further explored. Compared with the cell scratch at 0 h, cells in all groups migrated after 24 h, and the scratch was significantly reduced (Figure 6C,D). In the E_2_ and VD groups, the cell migration rates were 28.7% and 30%, respectively. Finally, the effects of estrogen and Vitamin D on the proliferation and adhesion gene expression of VK2/E6E7 cells were detected. After treatment with 1,25(OH)_2_D_3_, *PCNA* mRNA expression was increased (*p* < 0.05), but *Ki-67* mRNA did not change significantly. Vitamin D supplementation increased the expression of *E-cadherin* gene, and this result was similar to the results of animal experiments (Figure 4).

### 2.7. Effect of Vitamin D on Protein Expression in Rat Vaginal Tissues

**Figure 7:**
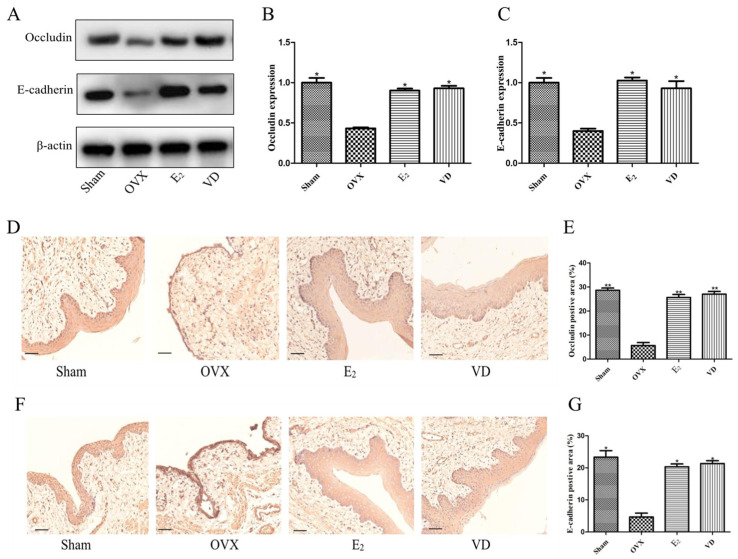
Western blot and immunohistochemical analysis of occludin and E-cadherin protein expression in vaginal epithelial tissue. (**A**) Western blot results of occludin and E-cadherin. Quantitative analysis of western blot image for occludin (**B**), E-cadherin (**C**). Immunohistochemical analysis of occludin protein (**D**,**E**). Quantitative analysis of E-cadherin protein (**F**,**G**). Scale bar = 100 µm. Data are the mean ± SD (n = 6). Compare with OVX group, * *p* < 0.05 and ** *p* < 0.01.

Having previously demonstrated by qPCR that Vitamin D can promote the transcription of adhesion genes in rat vaginal tissues (Figure 4), we further explored the expression of tight junction proteins in vaginal tissues by Western blot and immunohistochemistry. Western blot results showed that compared with the Sham group, the expression of occludin and E-cadherin proteins was significantly lower in the vaginal tissues of rats in the OVX group (Figure 7A). Supplementation with estrogen and Vitamin D significantly increased the expression of occludin and E-cadherin proteins, which promoted vaginal epithelial cell adhesion and vaginal tissue repair. Immunohistochemistry results showed that occludin protein in the Sham group was stained darker in the basal cell layer and protein expression was significantly increased (Figure 7D,E), and the proportion of positive cells reached 30%. cells in the OVX group were stained shallowly in the basal epithelial cell layer, and protein expression was low, accounting for only 5%. Compared with the OVX group, occludin protein expression was also significantly increased in the E_2_ and VD groups (*p* < 0.01). E-cadherin protein in the vaginal tissues of all groups also showed the same results (Figure 7F,G).

## 3. Discussion

Atrophic vaginitis is an inflammatory disease caused by the invasion and multiplication of pathogenic bacteria due to the decrease in oestrogen levels, atrophy of the vaginal wall and increase in pH value after menopause, resulting in a decrease in the number of *Lactobacilli* [24]. Thinning of the vaginal mucosa, congestion, and even superficial ulcers can be seen. Common symptoms include vaginal dryness, itching and difficulty in sexual intercourse [25]. It has serious adverse effects on the patient’s health as well as life. Systemic administration of oestrogen may cause adverse side effects. For example, it increases the risk of breast cancer, endometrial cancer, and cardiovascular disease [7]. Although topical oestrogen is safer, not all menopausal women can receive hormone supplementation. Therefore, the choice of non-hormonal replacement therapy for atrophic vaginitis is essential. In this study, atrophic vaginitis model was simulated by removing the ovaries of rats. The results showed that ovariectomised rats had a significant increase in vaginal pH, a significant decrease in uterine and vaginal weight, a decrease in the number of *Lactobacillus* in the vagina, and a decrease in the expression of PCNA, E-cadline and occludin proteins in the vaginal epithelium. Vitamin D supplementation could reduce vaginal pH, enhance PCNA expression in vaginal tissues, promote proliferation and keratinisation of basal epithelial cells, and improve vaginal and uterine atrophy. Immunohistochemical results showed that Vitamin D could increase the expression of E-cadherin and occludin proteins in vaginal tissues. It promotes the adhesion between vaginal epithelial cells and maintains the integrity of vaginal epithelium. In vitro experiments demonstrated that 1,25(OH)_2_D_3_ promotes proliferation, migration and adhesion of VK2/E6E7 vaginal epithelial cells. To our knowledge, this is the first demonstration that Vitamin D modulates the expression of tight junction proteins in vaginal tissues of ovariectomised rats. Vitamin D plays an important role in promoting the proliferation of vaginal epithelial cells, enhancing intercellular adhesion, and maintaining vaginal barrier integrity.

Vitamin D plays a key role in the growth and differentiation of squamous epithelial cells. Vitamin D exerts its biological role mainly by the binding of 1,25(OH)_2_D_3_ to VDR. Immunohistochemistry demonstrates that VDR localises to the basal and suprabasal layers of vaginal epithelial cells [11]. Mouse progenitors represent epithelial cells in culture whose terminal differentiation is regulated by 1,25(OH)_2_D_3_ [26]. Animal experiments demonstrated that Vitamin D combined with fluconazole ameliorates epithelial necrosis and ulceration of the vaginal wall, promotes regeneration of the vaginal epithelium, and acts as an adjuvant in vulvovaginal candidiasis (VVC) in mice [27]. Vitamin D also has an ameliorating effect on vaginal atrophy seen with tamoxifen in breast cancer patients. A randomised controlled trial demonstrated that Vitamins D and E vaginal suppositories were beneficial in improving vaginal atrophy, lowering vaginal pH, and improving vaginal maturation index in women with breast cancer treated with tamoxifen [28]. Lee et al., cultured the VK2/E6E7 cell line in starvation medium for 24 h, then added different concentrations of Vitamin D. Detection of cell viability revealed that 10^−8^ M Vitamin D was the optimal concentration for vaginal epithelial proliferation. It was further demonstrated that Vitamin D could stimulate the proliferation of vaginal epithelial cells through the activation of p-RhoA and Erzin by VDR [29]. The VDR was shown to have a beneficial effect in rat cell proliferation and differentiation of the vaginal epithelium had similar effects to E_2_ and did not increase endometrial thickness.

Tight junction (TJs) proteins play an important role in maintaining normal epithelial cell-to-cell adhesion and strengthening mechanical connections between cells [30]. Aberrant expression can lead to structural disruption and impaired function of epithelial cells. E-cadherin is a transmembrane, calcium-dependent cell adhesion protein that regulates cell-to-cell adhesion and maintains the structural and functional integrity of epithelial tissues [31]. Decreased expression of E-cadherin disrupts cell-to-cell contact, thereby allowing cells to migrate [32]. Vitamin D/VDR signalling maintains tissue barrier integrity by regulating tight junction components in multiple organs. Hussein et al., demonstrated that Vitamin D further maintains intestinal barrier function in a model of type 2 diabetes by up-regulating the expression of tight junction proteins and inhibiting inflammation [33]. Vitamin D supplementation in infants reduces the risk of suffering from intestinal damage by restoring tight junctions of ZO-1 and claudin 2 and decreasing the expression of TNF–α [34]. Zhang et al., demonstrated that chronic Vitamin D deficiency exacerbates TGF-1-mediated down-regulation of E-cadherin, which further leads to renal fibrosis and functional impairment [20]. Vitamin D induces the expression of occludin and claudin-14 proteins in the superficial umbrella cells of mature bladder and restores the integrity of the bladder epithelium in *E. coli* infected bladders [23]. Vitamin D, in addition to maintaining epithelial barrier function, improves the integrity of the vascular endothelial barrier by regulating vascular endothelial-calmodulin connections [35]. In this study, we found that Vitamin D increased the expression of E-cadherin and occludin proteins in the vaginal epithelial cells of ovariectomised rats, increased the barrier function of vaginal epithelial tissues, reduced invasion of pathogenic bacteria, and reduced inflammation.

Estrogen stimulates the production of glycogen by vaginal epithelial cells. *Lactobacillus* is able to break down glucose to produce lactic acid, which lowers vaginal pH. After menopause, the level of estrogen decreases in women, and the vaginal mucosa atrophies and bleeds in spots, and in severe cases, ulcers appear. This further leads to a decrease in *Lactobacillus*, invasion of exogenous pathogenic bacteria, and an imbalance in the vaginal flora, causing menopausal vaginal infections [36,37]. In addition to promoting the proliferation and differentiation of vaginal epithelial cells and maintaining the integrity of the vaginal barrier [38], Vitamin D enhances the antimicrobial response of the vaginal epithelium by regulating the immune function and influencing the type and distribution of vaginal flora [39]. A randomised controlled trial found a positive correlation between plasma 25(OH)D levels and *L. crispatus* abundance in the vagina in pregnant women taking 400 U of Vitamin D daily [40]. A cross-sectional study demonstrated that serum Vitamin D levels were negatively correlated with IL-6 in patients with recurrent vaginitis [41]. Vitamin D promotes the proliferation of vaginal epithelium, regulates vaginal flora and local immune environment, and is cheap and safer, making it a good choice for relieving symptoms of atrophic vaginitis.

## 4. Materials and Methods

### 4.1. Animals and Experimental Design

Female Sprague-Dawley rats of 10–12 weeks of age and weighing 150–200 g were purchased from Vital River Laboratory Animal Technology (Beijing, China). The rats were housed in a specific pathogen free (SPF) room (temperature: 22 ± 2 °C, dark light cycle for 12 h), during which they were allowed to drink and eat freely. After 1 week of acclimatization, rats were subjected to ovariectomy (OVX) or sham surgery (Sham), after which rats were given antibiotics for 3 days [42]. After ovariectomy, vaginal smears were performed to observe the estrous cycle of rats. Twenty-four rats were randomly divided into four groups (6 rats in each group): (1) Sham group, (2) OVX group, (3) estrogen group (E_2_): estrogen (Sigma-Aldrich Co., St. Louis, MO, USA) was dissolved in corn oil at a garage dose of 0.5 mg/kg per day for each ovariectomized rats, (4) Vitamin D group (VD): 1,25(OH)_2_D_3_ (Sigma-Aldrich Co., St. Louis, MO, USA) was dissolved in corn oil at a garage dose of 1000 U/kg per day per ovariectomised rat. The Sham and OVX groups were given equal amounts of corn oil. Vitamin D and estrogen were administered continuously for 2 months. At the end of the experiment, the vaginal pH of the rats was measured by pH test paper. After 12 h of fasting, anesthesia with sodium pentobarbital (150 mg per kg of body weight) was administered, followed by cervical dislocation. Serum was collected from the retro-orbital plexus after blood sampling. It was stored at −80 °C. Meanwhile, the uterus and vagina were immediately removed and weighed after observation. Finally, the tissues were stored at −80 °C for further analysis. This experiment was approved by the Institutional Animal Care and Use Committee (IACUC) of the Institute of Medical Laboratory Animals, Chinese Academy of Medical Sciences.

### 4.2. Serum Sex Hormone Level Testing

Serum sex hormone levels, including estrogen (E2), follicle-stimulating hormone (FSH) and luteinising hormone (LH), were measured by enzyme-linked immunosorbent assay (ELISA). The ELISA kits were purchased from Elabscience (Wuhan, China), and were used according to the manufacturer’s instructions.

### 4.3. Histological Analysis

Fresh uterine and vaginal tissues were cut and blood was rinsed with saline. The tissues were then soaked in 10% formalin buffer for 48 h for histological examination. The sections were stained with hematoxylin and eosin (H&E) and sealed with neutral resin. Scanning was performed under a 200× light microscope with a Nikon Eclipse 80i digital camera (Nikon, Tokyo, Japan). The thickness of vaginal and uterine mucosa was analysed using ImageJ software (NIH, Bethesda, MD, USA) (https://imagej.nih.gov/ij/, accessed on 10 August 2023).

### 4.4. RNA Isolation and Quantitative Real-Time PCR (qRT-PCR)

The ground vaginal tissue was used for gene expression analysis. Total RNA was extracted from vaginal tissues using TRIzol reagent (Invitrogen, Carlsbad, CA, USA). RNA concentration was determined using a Nanodrop 2000c spectrophotometer (Thermo Fisher Scientific, Waltham, MA, USA). Total RNA was reverse transcribed to cDNA using the primeScript RT reagent Kit (Takara, Otsu, Japan) according to the manufacturer’s instructions. qRT- PCR analysis was performed using the SYBR premix Ex Taq Kit (Takara, Otsu, Japan). Samples were analysed in triplicate. mRNA expression levels were normalised by *β-actin* and relative gene expression was determined using the 2^−∆∆Ct^ method. The primers were designed as follows: (presented in the sequence 5′-3′ forward, 5′-3′ reverse): *E-cadherin* (CGAGAGCTACACGTTCACGG, GGGTGTCGAGGGAAAAATAGG); *occludin* (GTCCGTGAGGCCTTTTGA, GGTGCATAATGATTGGGTTTG); *PCNA* (AAGGCTTCGACACATACCGC, AGCTGTACTCCTGTTCTGGGA); *Ki-67* (GTCTGTCTTACATGTTATTTAATC, ATCACCAATAAATAGTCTGGGCT); *β-actin* (CCTCACTGTCCACCTTCCA, GGGTGTAAAACGCAGCTCA).

### 4.5. Intravaginal Microbiological Testing

Vaginal microbiological tests were performed in ovariectomised rats after 2 months of oestrogen and vitamin D supplementation. A cotton swab moistened with saline was gently inserted into the vagina of the rats for about 0.5 cm, slightly rotated and removed, and the smear was evenly rotated on a slide and dried at room temperature. The number of *Lactobacilli* in the vaginal secretions was observed using Gram stain.

### 4.6. Cell Culture

Human vaginal mucosal epithelial cells (VK2/E6E7) were purchased from ATCC cell bank. After cell resuscitation, the cells were cultured in an incubator (51033775, Thermo Fisher Scientific, Waltham, MA, USA) at 5% CO_2_, 37 °C. DMEM (10313039, Gibco, Grand Island, NY, USA) medium containing 10% fetal bovine serum (10099141C, Gibco, Grand Island, NY, USA), 1% penicillin/streptomycin (Procell, Wuhan, China). When the cell density reached 80–90%, cell passaging was performed. Cells between 5–15 generations were used for experiments.

### 4.7. Cell Counting Kit 8 Assay

The optimal concentration of 1,25(OH)_2_D_3_ was determined using Cell Counting Kit-8 (CCK8) (Dongren Chemical Technology Co., Ltd., Shanghai, China). The VK2/E6E7 cell line was inoculated in 96-well plates and cultured for 24 h. Then it was treated with different concentrations of 1,25(OH)_2_D_3_ (10^−11^–10^−6^ M), respectively, and the blank control group was added with an equal amount of cell-free medium. After 24 h of incubation, 10 μL of CCK-8 solution was added to each well for dark incubation for 2 h, and then the absorbance value at 450 nm was measured by enzyme labelling instrument (VLBLATGD2, Thermo Fisher Scientific, Waltham, MA, USA).

### 4.8. Cell Migration Assay

Inoculate VK2/E6E7 cells in a 6-well culture plate. When the cells were spread over the culture plate, the cell monolayer was scratched using a 200 µL sterile pipette tip. The cells were washed three times with sterile PBS (Solarbio, Beijing, China) to remove the scratched cells and serum-free medium was added. Estrogen and Vitamin D were added in addition to the blank control group and cultured continuously for 24 h. Results were analysed using Image J software (https://imagej.nih.gov/ij/, accessed on 10 August 2023).

### 4.9. Immunofluorescence Staining

The cells in the culture plate were washed with PBS for 3 times, and then the slides were fixed with 4% paraformaldehyde for 15 min. Goat serum (Gibco, Grand Island, NY, USA) was added to the slides and closed at room temperature for 30 min, and a primary antibody was added to each slide and put into the wet box, incubated at 4 °C overnight. Then, add fluorescent secondary antibody and incubate in the wet box at room temperature for 1 h. Incubate with DAPI for 5 min, wash away the excess DAPI, seal the slides, and finally observe and collect the images under the fluorescence microscope.

### 4.10. Western Blot Analysis

Total protein was extracted from vaginal tissues ground into homogenates using RIPA lysis buffer (Solarbio, Beijing, China). protein sample concentrations were determined by BCA protein assay kit (Solarbio, Beijing, China). Electrophoresis was performed using a 10% SDS-PAGE gel. The gel was placed on a PVDF membrane activated with formaldehyde. 5% skimmed milk was closed for 1 h. The membranes were then incubated with the corresponding primary antibodies at 4 °C overnight. Finally, the membranes were soaked with secondary antibodies for 1 h at room temperature, and the washed PVDF was visualised using the ChemiDoc™ XRS system from Bio-Rad (Shanghai, China). The grey bands were quantified by Image J software (https://imagej.nih.gov/ij/, accessed on 10 August 2023). The primary antibody dilutions used in this study were: E-cadherin (1:1000, CST, 14472S), occludin (1:1000, CST, 68534S), β-actin (1:1000, CST, 93473SF).

### 4.11. Immunohistochemical Analysis

Paraffin sections of rat vaginal tissue were deparaffinized, hydrated and antigenically repaired using antigen repair solution. Drops of goat serum sealing solution were used for non-specific sealing for 10 min. next, drops of E-cadherin and occludin primary antibodies were added and placed in a wet box overnight at 4 °C, washed 3 times with PBS, drops of secondary antibodies were added, incubated for 30 min at room temperature, and washed 3 times with PBS. Finally, each section was stained with 100 uL of DAB (absin, china) solution, restained with hematoxylin, and finally dehydrated, sealed, and photographed.

### 4.12. Statistical Analysis

All data are expressed as mean ± standard deviation (mean ± SD) of at least three independent experiments. Student’s *t*-test was used for data analysis between two groups and one-way ANOVAs were used for data analysis between multiple groups. Graphpad prism 5.0 (La Jolla, CA, USA) was used for data analysis and graphics drawing. *p* < 0.05 were considered statistically different.

## 5. Conclusions

In conclusion, we found that Vitamin D supplementation promotes vaginal epithelial cell proliferation, improves vaginal atrophy, decreases pH, and does not increase endometrial thickness in ovariectomised rats. Vitamin D also reduced postmenopausal vaginitis by enhancing the expression of occludin and E-cadherin tight junction proteins, enhances intercellular adhesion, and regulating the vaginal microbiota in ovariectomised rats. Further studies are needed next to investigate the specific mechanism of action of Vitamin D in promoting the expression of tight junction proteins in vaginal epithelial cells.
